# *Mycobacterium abscessus* soft tissue infections associated with subcutaneous injection of lipolytic agents: An outbreak report and novel molecular epidemiology analysis approach

**DOI:** 10.14745/ccdr.v52i06a05

**Published:** 2026-06-30

**Authors:** Xavier Quan-Nguyen, Nicholas Waglechner, Maxime Veillette, Pier-Alexandre Vasil, Floriane Point, Bouchra Tannir, Melissa Zarandi-Nowroozi, Catherine Tsimiklis, Nadine Pétrin, Marty Teltscher, Anna Urbanek, Pierre-Marie Akochy, Joseph Cox, Robyn Lee, Simon Grandjean Lapierre

**Affiliations:** 1 Centre de Recherche du Centre Hospitalier de l’Université de Montréal, Université de Montréal, Montréal, QC; 2 Département de médecine, Université de Montréal, Montréal, QC; 3 Shared Hospital Laboratory, Toronto, ON; 4 Sinai Health System, Toronto, ON; 5 Dalla Lana School of Public Health, University of Toronto, Toronto, ON; 6 Direction régionale de santé publique, CIUSSS du Centre-Sud-de-l’île-de-Montréal, Montréal, QC; 7 Département de microbiologie, infectiologie et immunologie, Université de Montréal, Montréal, QC; 8 Hôpital du Sacré-Cœur-de-Montréal, Montréal, QC; 9 Centre hospitalier de l’Université de Montréal, Montréal, Canada; 10 Division of Infectious Diseases, Jewish General Hospital, Montréal, QC; 11 Laboratoire de santé publique du Québec, Montréal, QC; 12 Department of Epidemiology, Biostatistics and Occupational Health, McGill University, Montréal, QC; 13 Infectious Diseases and Immunity in Global Health Program, Research Institute of the McGill University Health Centre, McGill University, Montréal, QC; 14 McGill International TB Centre, McGill University, Montréal, QC; 15 Faculty of Medicine and Health Sciences, McGill University, Montréal, QC

**Keywords:** *Mycobacterium abscessus*, skin and soft tissue infection, mesotherapy, whole genome sequencing, molecular epidemiology

## Abstract

**Background:**

Management of *Mycobacterium abscessus* (*M. abscessus*) skin and soft tissue infections outbreaks require collaboration between clinicians, public health authorities and reference laboratories providing bacterial molecular identification and genotyping.

**Objective:**

This study reports on a *M. abscessus* skin and soft tissue outbreak linked to mesotherapy treatments in Montréal, Canada. We present an innovative approach combining Nanopore long read and Illumina short read whole-genome sequencing data with a novel open access bioinformatic molecular epidemiology pipeline to support public health investigations by identifying genomically related isolates.

**Methods:**

Public health investigations and physician questionnaires were used for outbreak identification and investigation. The complete genomes of six isolates from four individuals were sequenced. Nanopore and Illumina data were combined to assemble genomes *de novo* using a hybrid approach. Single nucleotide polymorphisms (SNPs) were identified within each genome by mapping the short reads to a reference genome. Single nucleotide polymorphisms distances among isolates, and between isolates and the reference genome were used alongside core SNP alignment analyses to evaluate genetic similarity between isolates and other contemporary *M. abscessus* genomes.

**Results:**

Identified individuals had received mesotherapy injections from the same esthetician within a one-month period. Outbreak isolates were genomically nearly identical, differing by only 0–2 SNPs when using the earliest collected isolate as a reference. A phylogenetic tree revealed genetic distinctness between outbreak isolates and other contemporary *M. abscessus* isolates from Montréal.

**Conclusion:**

Genomic sequencing and the presented bioinformatic approach can identify *M. abscessus* genomic relatedness suggestive of clinical outbreaks. This approach can support public health investigations, particularly when epidemiological links are uncertain.

## Introduction

Nontuberculous mycobacteria are ubiquitous organisms that can be found in healthcare and other environments and cause outbreaks of skin and soft tissue, pulmonary, lymph nodes, and joint infections (1–4). Diagnosis and treatment of *Mycobacterium abscessus* (*M. abscessus*) soft tissue infections is particularly challenging due their frequently atypical nodular and mildly inflammatory presentation, the inability of conventional bacterial cultures to identify this rapidly growing mycobacteria and its innate and acquired resistance to available antimicrobial agents (3,5). Outbreaks of *M*. *abscessus* skin and soft tissue infections have been previously reported in immunosuppressed individuals, following exposure to contaminated water, as post-operative complication of plastic surgery or following tattooing, acupuncture and mesotherapy treatments—a procedure in which multiple injections of pharmaceuticals or vitamins are delivered into the mesodermal layer of skin tissues to promote the loss of fat or cellulitis (5–7). Past outbreak investigations required coordinated efforts between public health, clinical professionals and reference laboratories providing bacterial molecular identification and genotyping (8,9).

The increased availability of next-generation sequencing platforms and the development of semi-automated data analysis pipelines support the increased uptake of next-generation sequencing-based molecular typing for outbreak investigation by public health and clinical microbiology laboratories (10). Bacterial whole-genome sequencing (WGS) allows much greater discrimination of epidemiologically clustered isolates and therefore helps support or refute the results of standard public health investigations (11–13). By combining WGS and epidemiological investigations, Olawoye *et al.* recently showed that health care-associated human-to-human transmission of *M. abscessus* was rare on the island of Montréal (14). In a multi-country study comparing strains from distinct global *M. abscessus* outbreaks, Tettelin *et al.* highlighted that while strains from the same outbreak were clustered together, high genomic relatedness could also be observed between epidemiologically unrelated isolates (15). Next-generation sequencing-based molecular typing systems must be well adapted to pathogens’ intrinsic genomic diversity and evolution to best complement public health investigations and accurately support or refute the relatedness of isolates.

We report on the investigation of a *M. abscessus* skin infections outbreak associated with mesotherapy procedures in Québec. We also describe a unique innovative approach combining long read and short read sequence data to improve the resolution of our molecular epidemiology analysis. This novel bioinformatic pipeline for the phylogenetic analysis of *M. abscessus* isolates is openly accessible.

## Methods

### Public health investigation

In 2021, an infectious disease clinician reported a case of disseminated panniculitis lesions associated with mesotherapy injections to the Montréal public health department. Although *M. abscessus* infections are not a mandatory reportable disease in Québec, the attending physician had perceived a potential risk for the public since aesthetic injections were the only identifiable risk factor. An investigation was initiated to identify and attempt to eliminate the infection source. An interview with the esthetician was conducted to catalog the injection products that were used and to review infection control practices. A detailed list of past clients having underwent mesotherapy procedures using the same injectable product and procedures was obtained. Putative outbreak cases were defined as individuals with microbiologically confirmed *M. abscessus* skin and soft tissue infections having been injected with the same product by the same esthetician. Cases were identified by reviewing the clients’ medical charts, including skin infections and positive culture results for *M. abscessus*. Attending physicians of all newly identified infected individuals were asked to complete a questionnaire collecting basic epidemiology, clinical presentation and microbiology data (**Appendix**, **Supplementary material**).

### Genomic sequencing and bioinformatic analysis

Mycobacterial isolates had initially been cultured by hospital-based clinical laboratories and referred to the *Laboratoire de santé publique du Québec* for *M. abscessus* speciation using 16s RNA sequencing. All available isolates from all individuals were retrieved and included in the molecular analysis. One randomly selected contemporary *M. abscessus* clinical isolate (control X) from Montréal was included as control for laboratory procedures and bioinformatic analyses. Mycobacterial DNA was extracted from pure culture and sequenced using Illumina and Oxford Nanopore next-generation sequencing platforms (Appendix, Supplementary material). All isolates passed sequencing quality controls including pre-alignment genomic coverage and absence of contaminating data (Appendix, Supplementary material).

Sequencing data was analyzed following previously described methods from Waglechner *et al.* (13). Both Nanopore and Illumina sequencing data were analyzed together using the Snakemake workflow engine (version 7.32.4) to execute the following pipeline using the ”hybrid” branch (16). Kraken2 v2.1.3, using a prebuilt MiniKraken database (version k2_plusfp_20220908_16) containing bacterial, viral, human, archaeal, vector, plasmid, protozoa and fungal sequences, was used for taxonomic assignment and contamination checking of each raw sequence data set (Nanopore and Illumina) for each sample (17,18). Using short read data, *de novo* assembly was performed and assigned to the most appropriate subspecies using mashtree along with reference sequences of *Mycobacterium tuberculosis* H37Rv (outgroup) (NC_000962) and the three *M. abscessus* subspecies: *M. abscessus* subspecies *abscessus* ATCC 19977 (NCBI GCF_000069185.1), *M. abscessus* subspecies *massiliense* CCUG 48898/JCM 15300 (NCBI GCF_000497265.2) and *M. abscessus* subspecies *bolletii* BD (NCBI GCF_003609715.1) (18). A hybrid *de novo* assembly was also prepared for each sample with long and short read data using dragonfly v1.2.1 with three rounds of short read polishing with polypolish v0.6.0 (19,20).

First, to assess outbreak individuals’ isolates relatedness, samples’ short read *de novo* assemblies for cases A through D were mapped against 1) the *M. abscessus* subspecies *massiliense* CCUG 48898 and 2) the hybrid short-read polished assembly of the putative outbreak earliest case (case B) using bwa-mem 2 v2.2.1 with default parameters (19). Single nucleotide polymorphisms (SNPs) were identified using samtools mpileup v1.14 (15). Variants were extracted into an alignment and subjected to recombination correction using gubbins v3.3.0 and counted using snp-dists v0.8.2 to assess relatedness of isolates (21). Core SNP alignments were analyzed by iqtree v2.3.6 with ultrafast bootstraps to examine the relatedness of outbreak isolates in the context of using either an outbreak isolate (case B hybrid assembly) or a subspecies type (*M. abscessus* subspecies *massiliense* CCUG 48898) as the reference genome. ETE (v3.1.1) was used to visualize maximum likelihood phylogenetic trees (22).

Second, to confirm outbreak isolates’ clustering within local circulating *M. abscessus* strains, a core genome was estimated from predicted open reading frames present in 99% or more of all assemblies from the outbreak isolates and 220 additional recent Montréal *M. abscessus* genomes from Olawoye *et al.* using the pangenome workflow from bactopia v3.1.0 using default parameters (14,23).

### Data availability

Whole genome sequencing data is available on the National Center for Biotechnology Information GenBank database (bioproject ID 1221978).

### Public health investigation

**Figure 1:**
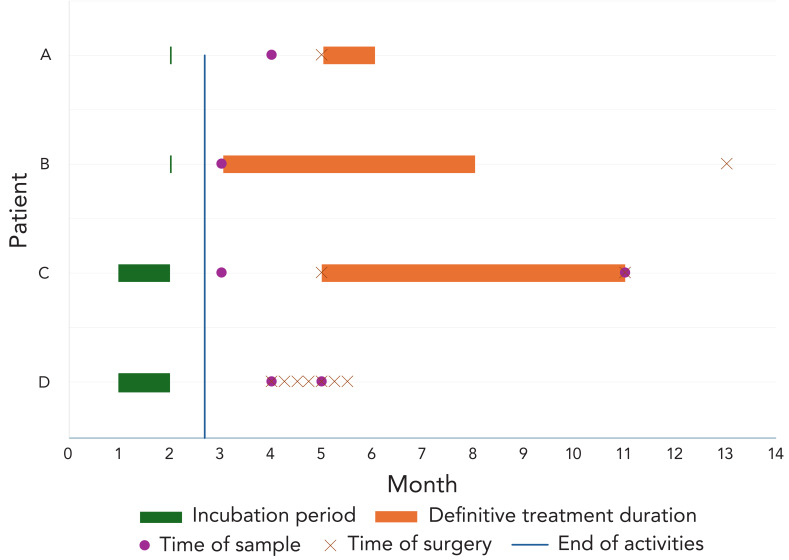
Montréal *Mycobacterium abscessus* outbreak timeline derived from medical records and attending clinical reports^a^ ^a^ Months are numbered based on the first clients’ injections (month 0). Incubation period refers to the time between injection and first reported symptoms. Definitive treatment duration refers to the length of *Mycobacterium abscessus* specific treatment following positive culture. Time of sample refers to the months in which the clinical samples were collected. Repeated sampling was performed for two individuals due to persistent skin lesions. Time of surgery refers to the months in which a surgical intervention was performed for the panniculitis lesions. End of activities refers to cessation of at risk injection treatments by the esthetician

The initial investigation included only the first two clients and revealed that an esthetician had performed mesotherapy injections in a rented commercial space. Esthetic services were advertised on social media as part of a spa package and were performed in a total of five clients during a one-month period. Among those, the public health investigation and medical chart review concluded that four clients were infected (**Figure 1**). Within days following the injection procedures, all four individuals had presented with subcutaneous, erythematous nodules at various sites where injections occurred, including the waist, lower back and thighs, accompanied by systemic symptoms (fever, chills, fatigue). Infected individuals had no other risk factors for mycobacterial infection, such as immunosuppression, implanted foreign material or other surgeries. Skin biopsy cultures had confirmed *M. abscessus* as the etiologic agent in all four individuals, including two with two distinct cultured isolates each (C–1 and C–2, D–1 and D–2). Each individual’s clinical management and treatment were retrieved (Appendix, Supplementary material).

A registered letter based on regulations in Québec’s public health law ordered the esthetician to assure a cessation of all activities. The esthetician had discarded all injection products after clients reported adverse reactions, so these products were unavailable for culture. The mesotherapy product lacked a Drug Identification Number in Health Canada’s Drug Product Database and was not a licensed natural product on Health Canada’s Licensed Natural Health Product Database (24,25). Health Canada’s Central Triage Unit, Regulatory Operations and Enforcement Branch was alerted to the possible *M. abscessus* contamination of the product. The case was referred to the Health Product Compliance East Unit of Health Canada which performed a compliance verification for the product; however, the results of the investigation, including additional information obtained from direct communication with the manufacturer, were not shared in accordance with the *Privacy Act*. As of February 2025, the Health Product Compliance East Unit reported “no more noncompliance” for the product. Also, no recalls or safety alerts have ever been issued for the product according to the Government of Canada’s Recalls and Safety Alerts database (26).

### Outbreak isolates relatedness molecular analysis

**Figure 2:**
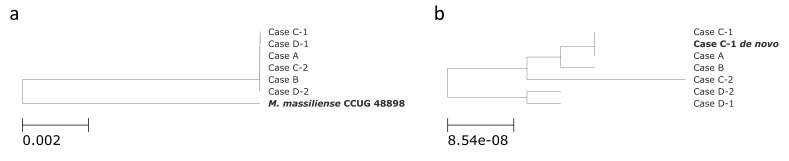
Maximum likelihood phylogenetic trees of Montréal *Mycobacterium abscessus* outbreak isolates^a^ Abbreviation: *M. massiliense*, *Mycobacterium abscessus* subspecies *massiliense* ^a^ Core genome single nucleotide polymorphism (SNP) alignments were produced by mapping short read data against a) the *M. abscessus* subspecies *massiliense* CCUG 48898 reference genome and b) a *de novo* long read assembly of an early outbreak case polished with short read data. Reference sequences used for mapping in each tree are indicated in bold

**Figure 3:**
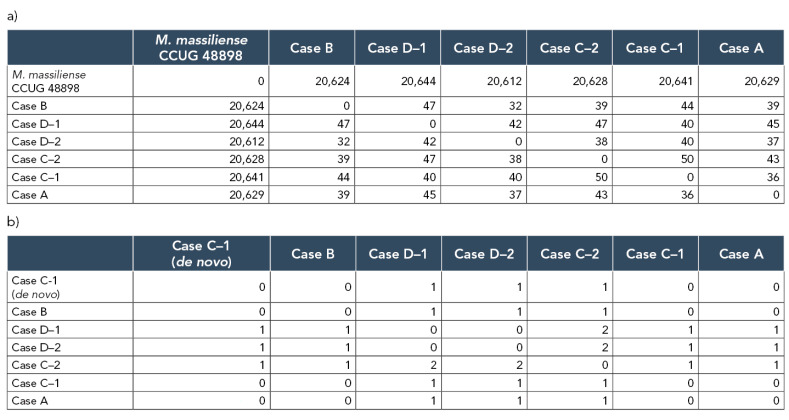
Outbreak isolates pairwise^a^ single nucleotide polymorphisms distance matrices Abbreviation: *M. massiliense*, *Mycobacterium abscessus* subspecies *massiliense* ^a^ Pairwise distances using a) Illumina data mapped against *M. massiliense* CCUG 48898, b) Illumina data mapped against *de novo* long read assembly polished with Illumina data against an early outbreak case

All outbreak-related isolates were identified as *M. abscessus* subspecies *massiliense*. The contemporary clinical control isolate was identified as *M. abscessus* subspecies *abscessus* and excluded from further pairwise SNP comparisons using the outbreak-specific hybrid assembly reference. Short read data from the outbreak isolates mapped against a polished assembly of an early outbreak isolate (Case B) showed 100% mapping coverage. The outbreak isolates were nearly identical with each other by pairwise SNP distance, ranging from 0 to 2 SNPs. The same procedure using *M. abscessus* subspecies *massiliense* CCUG 48898 as a reference yielded pairwise SNP distances ranging from 32 to 50 SNPs between outbreak isolates, while outbreak isolates ranged from 26,612 to 26,644 pairwise SNPs apart from the reference (**Figure 2**, **Figure 3**). Sequentially cultured clinical isolates from the same individual (e.g., C–1 vs. C–2, D–1 vs. D–2) sampled up to eight months apart were not more closely related to the earliest isolate in each pair than to isolates from other individuals.

### Outbreak isolates clustering amongst locally circulating strains

**Figure 4:**
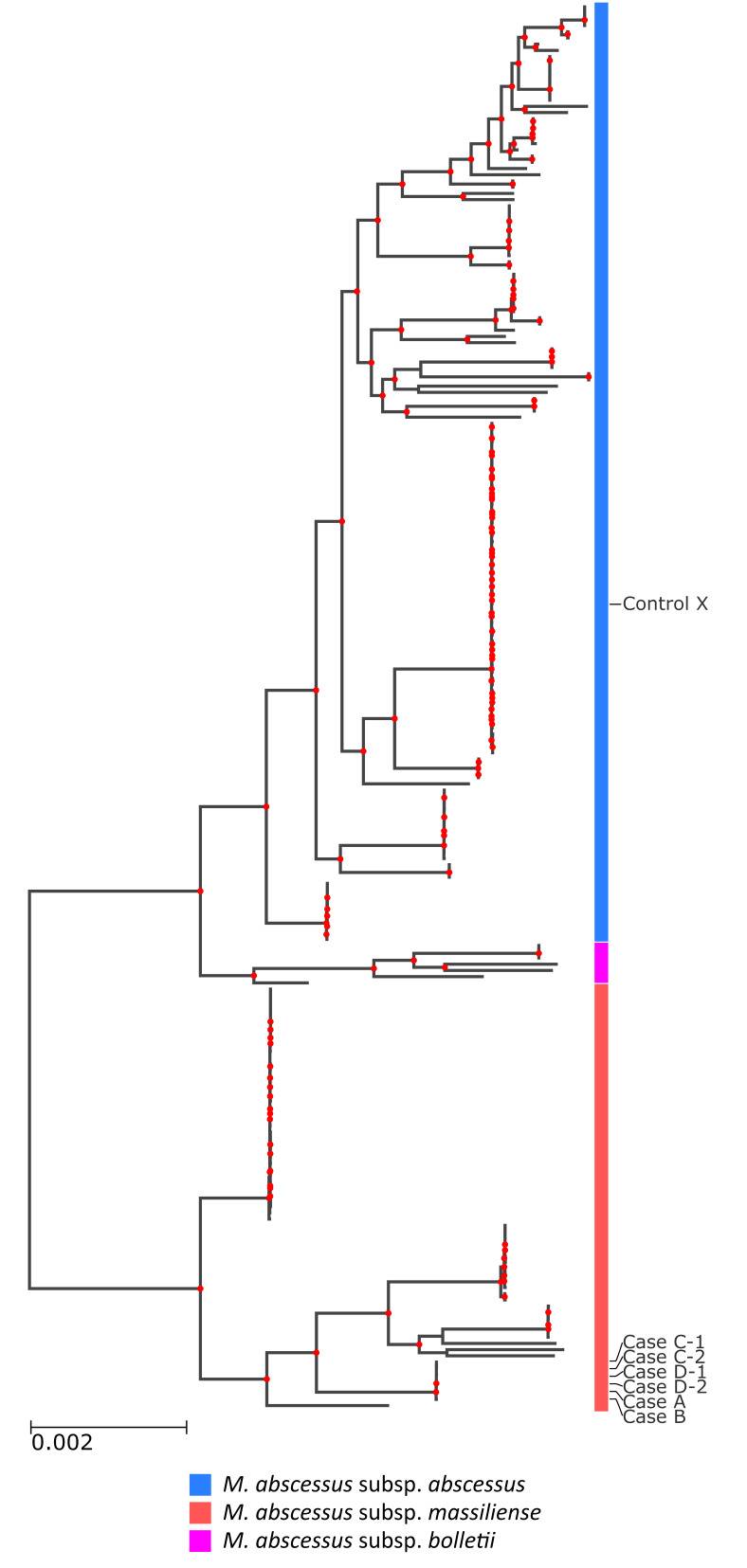
Core-genome single nucleotide polymorphisms phylogeny of *Mycobacterium abscessus* isolates from Montréal in 2025^a^ Abbreviations: *M. abscessus*, *Mycobacterium abscessus*; subsp., subspecies ^a^ The core genome was estimated from open reading frames of the assembled outbreak isolates along with 220 additional isolates from the island of Montréal. *Mycobacterium abscessus* subsp. *massiliense* isolates cultured from the skin infection outbreak individuals exhibit high relatedness compared to the Montréal *M. abscessus* catalog

The pangenome was constructed using assemblies from the six outbreak isolates as well as the Control X isolate along with 220 additional *M. abscessus* isolates collected from 2010 to 2018 recently published from the island of Montréal and other Québec laboratories (5). The core genome (sequences found in 99% or more of genomes) consisted of 3,403 open reading frames totaling 3,951,644 bp when aligned. After recombination detection and masking with ClonalFrameML, pairwise SNPs were counted from this alignment and used to produce a phylogenetic tree of 227 Montréal *M. abscessus* isolates from all three subspecies (**Figure 4**). This tree shows that the outbreak isolates are distinct from other subspecies *massiliense* isolates previously sequenced except for one. This isolate had been cultured from the respiratory track of a cystic fibrosis patient who had not received mesotherapy treatments and shared no epidemiological links with the outbreak individuals.

## Discussion

During this *M. abscessus* infection outbreak investigation, we confirmed the ability of a conventional epidemiological investigation to comprehensively identify individuals involved in a point-source outbreak. We also developed an improved molecular approach leveraging both short (Illumina) and long (Nanopore) read sequence data to confirm the relatedness of epidemiologically linked cases and assess their bacterial isolates’ molecular clustering within an extended catalog of locally circulating strains.

Esthetician services and easy access to injection products on-line do not fall under Health Canada regulations. In Québec, anyone who wishes to receive regulated product injections for cosmetic purposes must undergo a prior medical evaluation so that a doctor can establish an individualized treatment plan. A nurse or licenced practical nurse, if prescribed to do so, may undertake such treatments. Since esthetician services are not regulated, it is important for the public to be aware of the risks associated with invasive treatments which should only be provided under medical supervision. In this context, we highlight the importance of case signaling by health professionals to Public Health departments. This outbreak investigation was initiated following physicians’ report of a post-injection infection. Public health authorities have a responsibility to investigate real and perceived threats to the public’s health; as seen in this investigation, they use their authority under the law to order the cessation of activities or use of suspected products, until a public health investigation can be completed and the threat removed or diminished.

A strength of our investigation is the combination of traditional epidemiological investigation and multi-modal sequencing molecular epidemiology. Using clinically related isolates from this outbreak investigation, we extended our previously published *M. abscessus* bioinformatic pipeline to make use of long read data from Oxford Nanopore instruments (15). Long read data enabled the production of essentially closed and complete genome assemblies of the outbreak isolates and enabled the use a more relevant reference for pairwise SNP analysis. Before the common availability of long read sequencing, mapping relied on using the closest publicly available genome sequence that, in practice, could result in bias in SNP calling. This bias can be seen when mapping our outbreak isolates against the *M. abscessus* subspecies *massiliense* CCUG 48898 strain where the clinical isolates were collectively approximately 28,000 SNPs apart from the reference. More importantly, bias was introduced at the mapping and SNP calling stages that exaggerated the differences between outbreak isolates. Using the complete *de novo* Case C–1 assembly as a reference 100% of the reference was covered revealing limited genomic variability between outbreak isolates where each was within 0–2 SNPs of each other.

In this study, all outbreak isolates were found to be almost genetically identical. Sequentially sampled isolates from the same infected individuals did not exhibit higher or lower genomic relatedness (e.g., C–1 and C–2, D–1 and D–2). Previous studies suggested that *M. abscessus* subspecies *massiliense* has a lower mutation rate than other *M. abscessus* (27–29). In a previous *M. abscessus* molecular epidemiology study conducted over nine years, clinical isolates collected from the same individual did preferentially clustered together (14). Bryant *et al.*, suggested that SNP distances of less than 25 were associated with related *M. abscessus* subspecies *massiliense* isolates and that SNP distances of 50 to 200 correlated with distinct clusters (27). Doyle *et al.* found median SNP distances of 2,084 within the same sequence cluster and advocated for variant calling against more genetically similar reference sequences or when unavailable, the first isolated sample, to better highlight differences between strains and decluster genetically similar sequences (30). We believe the differences between within-host bacterial evolution in lungs and soft tissue infections, the bioinformatic analytic approach and the choice of alignment reference sequence explain these differences between previously published results and ours. Among other previously reported *M. abscessus* skin and soft tissue outbreaks, one included short-read sequencing-based WGS analysis and also found related isolates to be within three SNPs of each other and source environmental isolates (6).

## Conclusion

Nontuberculous mycobacteria skin infections outbreaks can be associated with aesthetic injection products. Bacterial WGS and the presented bioinformatic pipeline can support *M. abscessus* outbreak investigations. Combining Nanopore and Illumina sequencing data for molecular clustering analyses represents a novel approach to improve *M. abscessus* clinical isolates clustering analysis. Genomic sequencing is of important value when molecular clustering further supports a clinically suspected iatrogenic infectious.

## Appendix

Supplemental material is available upon request to the author: simon.grandjean.lapierre@umontreal.ca

Figure S1: Data collection questionnaire

Figure S2: Mycobacterial DNA extraction and sequencing

Figure S3: Sequencing quality metrics

Figure S4: Clinical management and treatment

Table S1: *Mycobacterium abscessus* outbreak patients’ treatment and clinical management
